# Learning Design Rules for Selective Oxidation Catalysts from High-Throughput Experimentation and Artificial Intelligence

**DOI:** 10.1021/acscatal.1c04793

**Published:** 2022-01-31

**Authors:** Lucas Foppa, Christopher Sutton, Luca M. Ghiringhelli, Sandip De, Patricia Löser, Stephan A. Schunk, Ansgar Schäfer, Matthias Scheffler

**Affiliations:** † The NOMAD Laboratory, Fritz-Haber-Institut der Max-Planck-Gesellschaft, Faradayweg 4-6, D-14195 Berlin, Germany; ‡ The NOMAD Laboratory, Humboldt-Universität zu Berlin, Zum Großen Windkanal 6, D-12489 Berlin, Germany; # FAIRmat, Humboldt-Universität zu Berlin, Zum Großen Windkanal 6, D-12489 Berlin, Germany; § BASF SE, Carl-Bosch-Straße 38, D-67065 Ludwigshafen, Germany; ∥ hte GmbH, Kurpfalzring 104, D-69123, Heidelberg, Germany

**Keywords:** artificial intelligence, subgroup discovery, high-throughput experimentation, selective oxidation, propylene, ruthenium

## Abstract

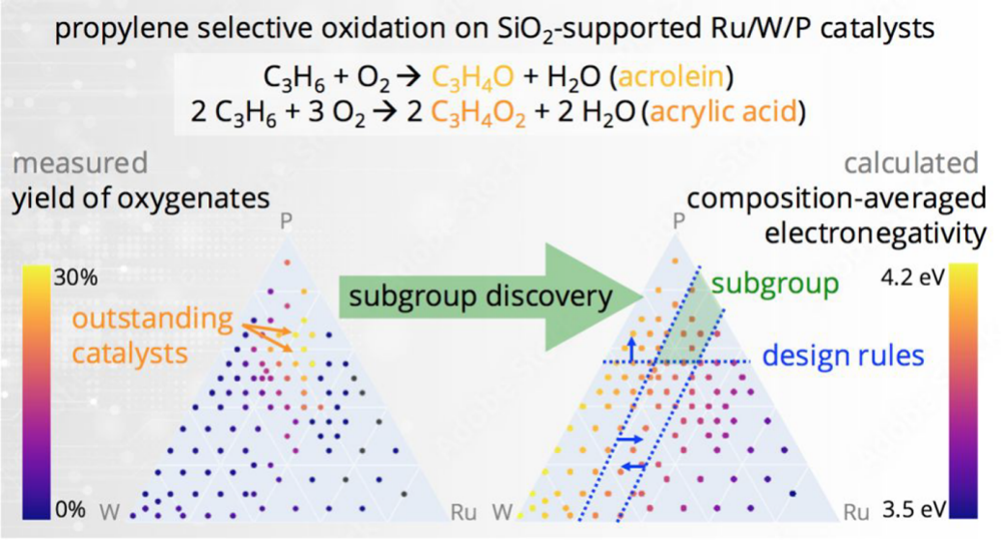

The design of heterogeneous catalysts is challenged by the complexity of materials and processes that govern reactivity and by the fact that the number of good catalysts is very small in comparison to the number of possible materials. Here, we show how the subgroup-discovery (SGD) artificial-intelligence approach can be applied to an experimental plus theoretical data set to identify constraints on key physicochemical parameters, the so-called SG *rules*, which exclusively describe materials and reaction conditions with outstanding catalytic performance. By using high-throughput experimentation, 120 SiO_2_-supported catalysts containing ruthenium, tungsten, and phosphorus were synthesized and tested in the catalytic oxidation of propylene. As candidate descriptive parameters, the temperature and 10 parameters related to the composition and chemical nature of the catalyst materials, derived from calculated free-atom properties, were offered. The temperature, the phosphorus content, and the composition-weighted electronegativity are identified as key parameters describing high yields toward the value-added oxygenate products acrolein and acrylic acid. The SG rules not only reflect the underlying processes particularly associated with high performance but also guide the design of more complex catalysts containing up to five elements in their composition.

## Introduction

Heterogeneous catalysis is governed by an intricate interplay of multiple processes^1^ such as the surface reaction networks and the typically unknown dynamic restructuring of the catalyst material under the reaction conditions. Thus, the design of new materials is challenging. While theoretical approaches attempt to address the complexity of heterogeneous catalysis,^2^ the explicit atomistic modeling of the full catalytic progression by first-principles methods is impractical. Another approach for identifying novel catalysts consists of the use of high-throughput experimentation (HTE) to test large numbers of materials.^3^ However, utilizing the information obtained by the experiments to decide on the next promising materials to investigate is not straightforward.^4^ As the number of possible materials is practically infinite and the number of good catalysts is very small, the direct approach is unlikely to identify the needed catalyst material.

First, when large libraries of materials are tested, the detailed characterization of each material is typically not feasible. Thus, only a small amount of information on the structure and physicochemical properties of each compound might be available. This hinders an in-depth understanding of the underlying processes governing reactivity, which could be used for rational catalyst design. Second, distinct catalytic mechanisms might operate, depending on the materials and reaction conditions, and only very few situations result in good catalytic performance. This leads to an unbalanced distribution between high- and low-performance scenarios and brings into question the usefulness of *global* models to help deciding on the next materials to be tested. These models are trained to describe all materials and reaction conditions simultaneously by minimizing the expected average prediction error over all samples. While this approach may provide an accurate prediction on average, it does not necessarily allow for a focused modeling of the most interesting materials and mechanisms. Alternative approaches for catalyst design are therefore required.

Several recent studies have described artificial-intelligence approaches based on physicochemical parameters for the analysis and discovery of catalytic systems.^5^ Here, we apply the subgroup-discovery (SGD) artificial-intelligence local approach^6^ to a hybrid data set obtained from HTE and theory to identify key physicochemical descriptive parameters and constraints on their values, i.e., rules, which are particularly associated with high performance. The reactivity measured by HTE is used as a target in the SGD analysis. The temperature- and composition-dependent physicochemical properties evaluated with density functional theory (DFT) calculations are used as candidate descriptive parameters.

**Figure 1:**
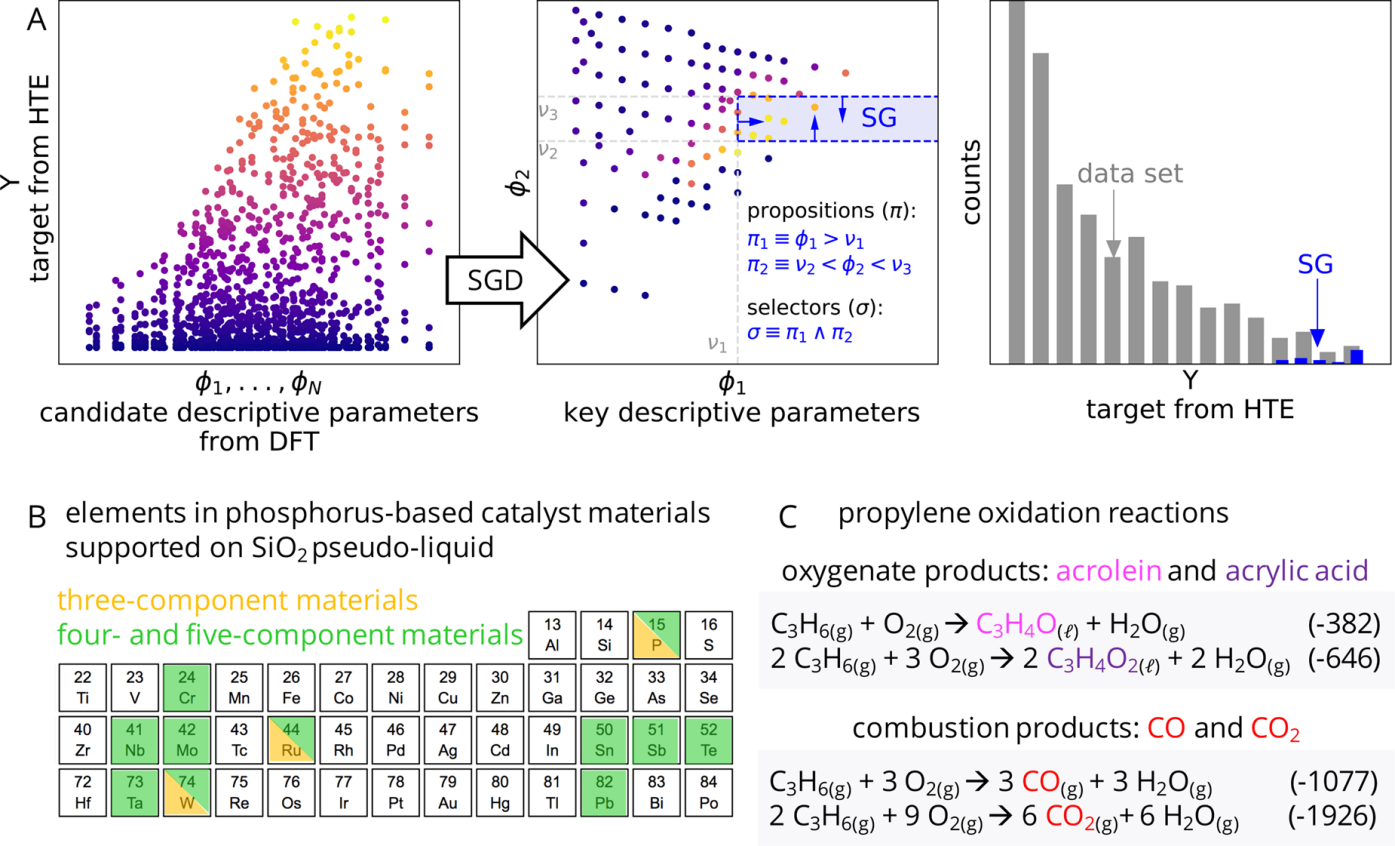
(A) SGD approach for identifying key descriptive parameters and rules associated with materials and reaction conditions with outstanding catalytic performance. The rules are given by the propositions and consist of constraints on the values of key descriptive parameters. “∧” denotes the “AND” connector. (B) Elements entering the composition of the SiO_2_-supported materials. (C) Competing reactions in propylene oxidation leading not only to the desired oxygenates but also to the combustion byproducts. The values shown in parentheses in (C) are the reaction enthalpies, in kJ/mol.^9^

The SGD approach has been applied in computational catalysis^7^ and materials science.^6e,8^ It starts with the generation of a pool of propositions (π), statements about the data that apply only to a portion of the data set. For the case of continuous candidate descriptive parameters, the propositions are inequalities constraining their values. Then, SGD identifies selectors (σ), i.e., statements formed by a number of propositions and the “AND” connector (denoted “∧”), that result in the selection of subgroups of materials and conditions with the most outstanding distributions of the target values with respect to the whole data set (Figure 1A). The propositions entering these selectors can be seen as rules describing the exceptional SG behavior. The parameters entering these propositions are in turn the key, most relevant descriptive parameters, out of all the offered parameters, associated with the desired reactivity. Because the SG search is performed by maximizing a quality function that measures how outstanding specific subselections of data points are, this approach identifies a local behavior. Thus, the identified rules reflect the specific underlying processes resulting in outstanding performance.

We apply the SGD-HTE and theory approach to the selective oxidation of propylene on SiO_2_-supported catalysts based on ruthenium, tungsten, and phosphorus. By using the product yield measured by HTE as a target, we circumvent the need for the explicit modeling of the full catalytic progression. Additionally, because the candidate descriptive material parameters can be calculated by first-principles methods, extensive material characterization by experiment is not required and the resulting SG rules can be used to identify promising catalyst candidates which have not yet been synthesized by experiment.

## Selective Oxidation Reaction and High-Throughput Experimentation

The selective partial oxidation of light alkanes to value-added olefins or oxygenates is an efficient route for feedstock upgrading.^10^ However, the intricate surface reaction networks^11^ typically lead to product mixtures containing up to 20 different molecules, including undesirable byproducts such as CO and CO_2_. In order to selectively produce the olefins or the oxygenates, mixed-metal oxide or phosphate heterogeneous catalysts based on molybdenum and vanadium redox-active species have been used, such as MoVTeNbO_*x*_ and the state of the art industrial catalyst for *n*-butane selective oxidation, vanadyl pyrophosphate. Several recent investigations have explored the physicochemical properties and the catalytic activity of mixed-metal phosphates in a systematic way.^12^

Platinum-group-metal-based catalysts commonly result in hydrocarbon combustion products. However, ruthenium-based materials also catalyze the partial oxidation of methane to CO.^13^ Moreover, the isolation of ruthenium species was proposed as a strategy to increase the catalyst selectivity in oxidation reactions.^14^ Analogously, it was shown that the isolation of vanadium species in a tungsten phosphate matrix increases the catalyst selectivity toward oxygenates in the *n*-butane oxidation reaction.^15^ In this study, we investigate materials based on ruthenium combined with tungsten and phosphorus (Figure 1B) as an alternative class of catalysts for selective oxidation. The combination of Ru with tungsten and phosphorus, in a tungsten phosphate like matrix, could favor selectivity toward the desired olefins and oxygenates, following a catalyst design strategy based on the dilution of highly active metal sites. With the aim of studying these systems, HTE measurements were performed using 120 different *three-component* catalyst compositions containing ruthenium, tungsten, and phosphorus in different proportions. At each catalyst composition, several reaction temperatures between 200 and 400 °C were examined. The detailed preparation, characterization, and reactivity analysis of these catalysts in the selective oxidation of *n*-butane, propane, and propylene is discussed in a separate contribution.^16^ In this paper, we only provide details of the propylene selective oxidation reaction (Figure 1C).

All of the reactions were carried out in tubular, fixed-bed reactors with the following reaction feed: Ar, H_2_O, N_2_, O_2_, and propylene (C_3_H_6_) with molar rates of 4.015, 4.015, 104.40, 20.08, and 1.57 mmol/h, respectively. The same mass of catalyst was used in all reactions, so that the contact time, in terms of volumetric flow per mass of catalyst, was kept fixed across experiments. These three-component catalysts were prepared on a SiO_2_ pseudoliquid support and might present a disordered, possibly amorphous, structure. The atomic structures of all the tested catalysts are not known in detail. However, similar catalytic performance was found for crystalline and disordered phases at the same composition.^16^ This indicates that the composition is more crucial for the catalytic performance than the degree of crystallinity.

In HTE, a large materials space is accessible for catalyst design by changing the relative amount of each component and the specific elements on the catalyst composition. Approaches to guide the efficient exploration of such a materials space, indicating the most promising compositions to be tested next, are thus desirable. The most interesting compositions are those that display both considerable activity, i.e., those providing significant propylene conversion, and selectivity, i.e., those that specifically form the desired oxygenates (acrolein and acrylic acid, Figure 1C) from propylene. This is motivated by using the yield of oxygenates *Y*_oxygenates_ as target in our SGD analysis, defined as
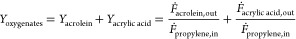
1In eq 1, *Ḟ*_A__,in_ and *Ḟ*_A__,out_ denote the molar rate, in mmol/h, of species A in the reactor feed and outlet, respectively. Our goal is to identify key parameters and rules describing materials and reaction conditions that give high yields of oxygenates.

## Subgroup Discovery Approach

The two main crucial aspects in SGD are the offered candidate descriptive parameters and the quality function. In this work, we use the reaction temperature (*T*) and the phosphorus molar content (*x*_p_) as experimental candidate parameters. In addition, we include a set of free-atom properties as candidate descriptive parameters to characterize the catalyst material in terms of the proportion and chemical nature of the elements entering the composition. The following elemental properties are used:

- the radii of maximum electron density of *s*, *p*, *d*, and valence orbitals (*r*_*s*_, *r*_*p*_, *r*_*d*_, and *r*_val_, respectively)
- the Kohn–Sham single-particle eigenvalues of the highest occupied and lowest unoccupied states (ε_H_ and ε_L_, respectively)
- the electron affinity (EA)
- the ionization potential (IP)
- the electronegativity (EN), defined as .

These properties were calculated for the isolated atoms using DFT-PBEsol^17^ and the FHI-aims^18^ code (further calculation details and values for the elemental properties used in the work are available in the Supporting Information). *r*_val_ is defined as the radius of the highest occupied state. For a given catalyst composition, the per-element free-atom properties are converted into system-specific properties by taking the composition-weighted average
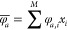
2where *φ*_*a*_ is an arbitrary elemental property, *x*_*i*_ is the molar content of element *i* in the material, and *i* runs over all *M* elements in the composition. For the three-component materials, *M* = 3. We note that oxygen is also present in all materials, but its proportion is not known from the catalyst formulation nor measured for all materials. Therefore, the oxygen content is not included in the material’s characterization. Properties that can be readily calculated for the free atom are advantageous to structure-based properties because they do not have to be re-evaluated for each new material. Furthermore, it should be noted that the composition-weighted average is defined for an arbitrary number of components (or elements). Therefore, the key descriptive parameters identified by the SGD of three-component materials can be used to design materials containing different elements or more than three components (*vide infra*). This would not be the case if only composition parameters (e.g., *x*_P_, *x*_Ru_, and *x*_W_) were used, since these quantities are not defined for materials containing elements different from Ru, P, and W or more than three components. In total, 11 descriptive parameters are used in our SGD analysis: *T*, *x*_P_, *r̅*_*s*_, *r̅*_*p*_, *r̅*_*d*_, , , , , , and .

As the SGD quality function, we use
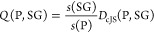
3where the *coverage* is the ratio between the number of data points in the subgroup, *s*(SG), and the total number of data points in the whole data set, *s*(P), and *D*_cJS_(P,SG) is the cumulative Jensen–Shannon divergence between the distribution of the target values in the SG and the distribution of the target values in the whole data set.^19^ The coverage term controls the subgroup size and prevents very small SGs with little statistical significance from being selected. The second term, *D*_cJS_, is the cumulative-distribution-function formulation^19^ of the Jensen–Shannon divergence, which is a properly symmetrized version of the information-theoretical Kullback–Leibler divergence. *D*_cJS_ measures the dissimilarity between two distributions: it assumes close to zero values for similar distributions and increases, for instance, as the distributions have different standard deviations or different means. Thus, the second term in eq 3 favors the identification of SGs presenting target values as “unusual” as possible in comparison to the majority of the observations. It also favors distributions that are contained in narrower value ranges in comparison to the whole data set. When most of the data points at hand contain low-performing materials and conditions, the use of *D*_cJS_ in the quality function allows focusing on the exceptionally high performing materials.

The SGD approach contrasts with conventional artificial-intelligence methods such as decision trees, which are based on the optimization of a function that measures the global performance of the model across the whole data set (e.g., mean absolute error or root mean squared error). While global approaches may provide a good description on average, they do not focus on the outstanding data points. We also note that SGD identifies the key, most relevant descriptive parameters out of many candidate descriptive parameters. Conversely, conventional regression approaches (e.g., multivariate regression) exploit all of the parameters simultaneously. As a consequence, the most important parameters are not necessarily determined by such an analysis. This is problematic when obtaining the candidate descriptive parameters for new materials (extrapolation) involves significant experimental or computational effort. Further SGD details, including a detailed description of the approach and of the Jensen–Shannon divergence are available in Supporting Information. SGD was compared to the decision-tree approach in previous works by some of us.^7^

## Subgroup of Three-Component Catalysts with Exceptional Performance

**Figure 2:**
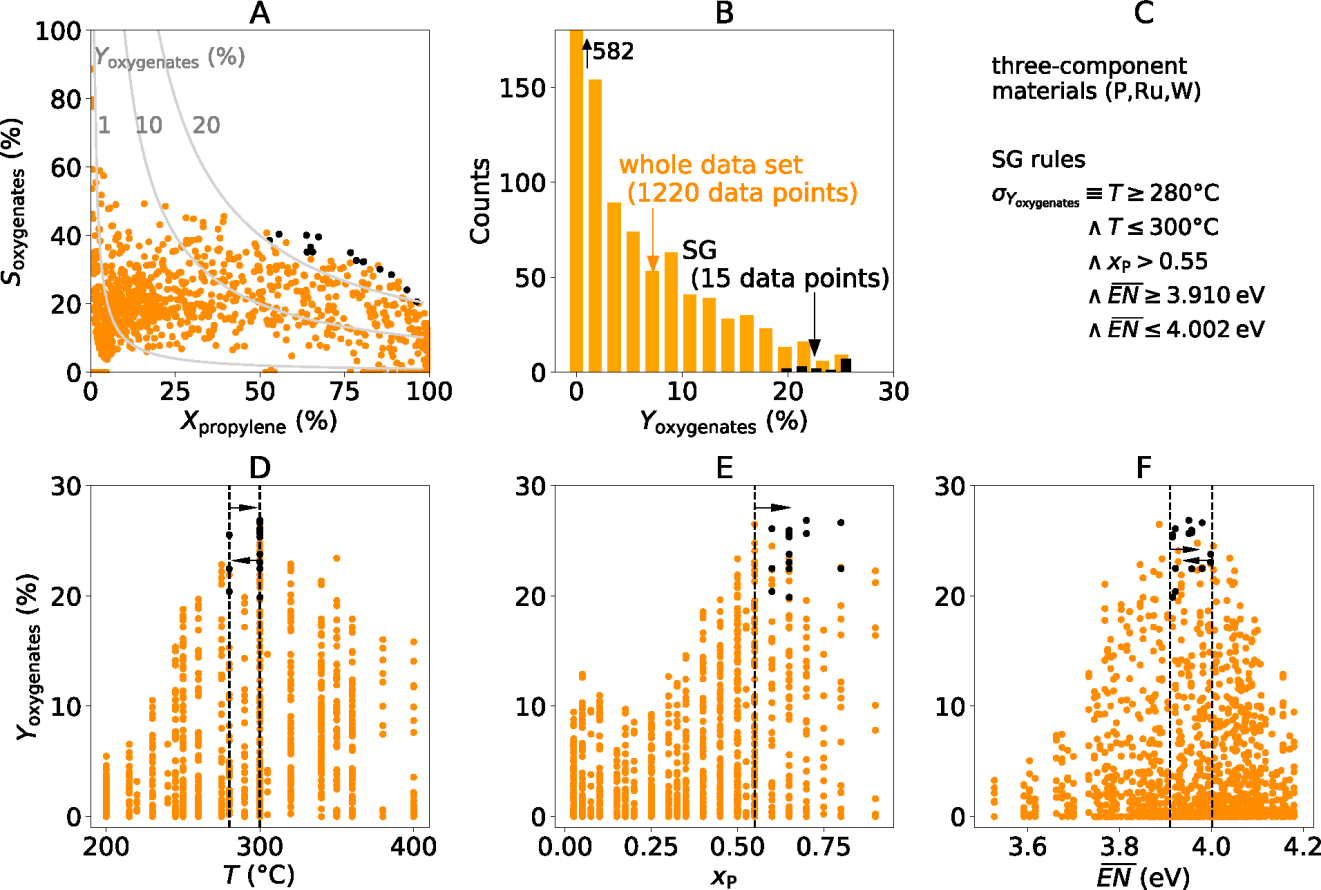
SGD analysis of propylene selective oxidation using three-component materials with ruthenium, tungsten, and phosphorus: (A) overview of reactivity measured by HTE; (B) distribution of oxygenate yield over the data set of 1220 measurements; (C) identified rules describing the SG; (D–F) SG rules (indicated by the black dashed lines and arrows) on the identified key descriptive parameters temperature (*T*), phosphorus molar content (*x*_P_), and composition-averaged electronegativity (), respectively. The data points corresponding to the identified SG are displayed in black. The propylene conversion and the selectivity toward oxygenates are defined by  and , respectively.

The propylene conversion vs oxygenates selectivity profiles and the distribution of yield of oxygenates in the data set (Figure 2A,B, respectively) show that the vast majority of observations correspond to low performance. Indeed, 50% of the measured materials and conditions result in a less than 2% oxygenate yield and only 41 measurements, out of 1220, are associated with yields of oxygenates above 20%. The average oxygenate yield over the whole data set is equal to 4.83%, and the maximum *Y*_oxygenates_ value is 26.85%.

By applying the SGD, we identified several SGs providing near-optimal quality-function values (Figure S3). Among the SGs displaying quality-function values within 40% of the optimal value, we selected, for further analysis and discussion, the SG that presents the highest value of cumulative Jensen–Shannon divergence (0.693). This SG contains only 15 data points, i.e., approximately 1.2% of the data set, which all have high yields of oxygenates (Figure 2A,B, in black). The average yield of oxygenates in this SG is equal to 24.15%: i.e., 5 times higher than the average on the whole data set. This SG is described by rules on three descriptive parameters: 280 ≤ *T* ≤ 300 °C, *x*_P_ > 0.55, and  (Figure 2C).

**Figure 3:**
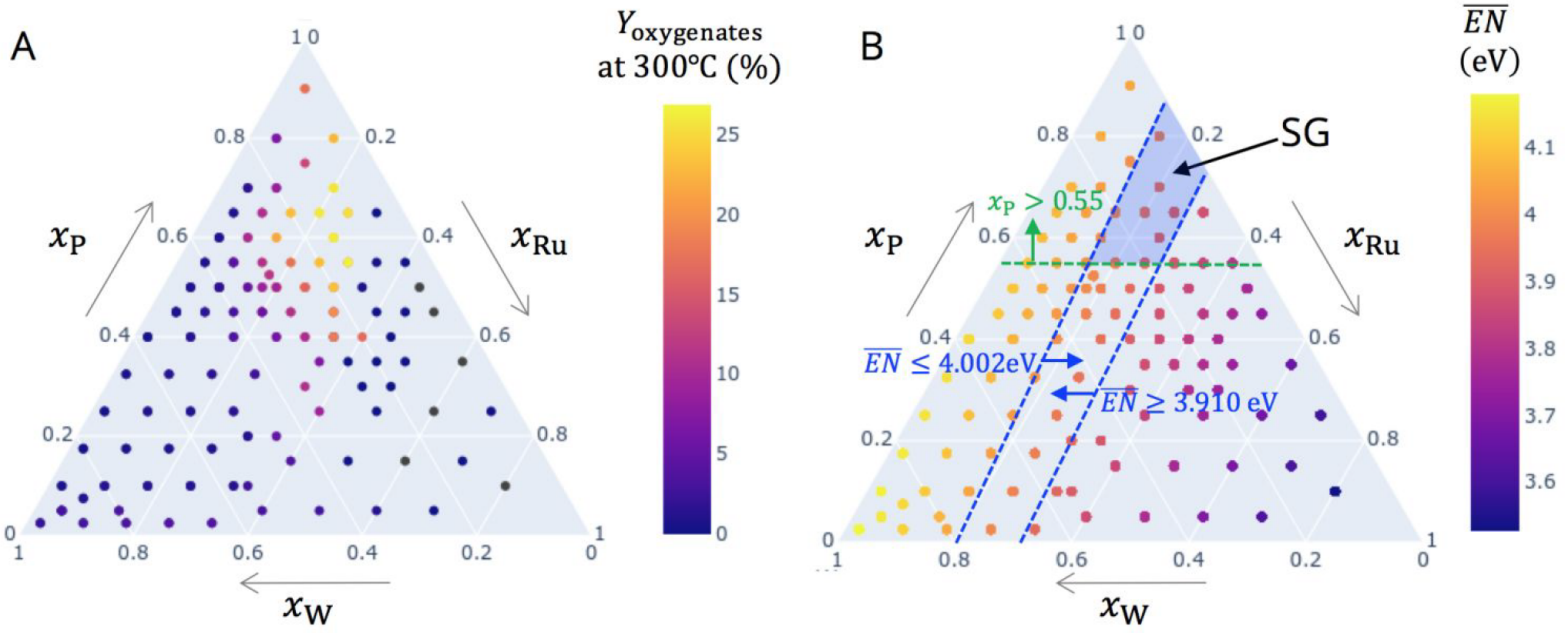
Ternary diagrams for three-component materials with ruthenium, tungsten, and phosphorus tested in propylene selective oxidation using HTE: (A) measured yield of oxygenates at 300 °C; (B) composition-averaged electronegativity  for each tested composition. The SG rules are shown by the dashed lines and arrows in (B), and the portion of the ternary diagram selected by the SG rules is shown in blue.

The rule on the temperature highlights that the highest yields of oxygenates are observed for intermediate temperatures within the tested range of 200–400 °C (Figure 2C). This could be related to the fact that the yield of oxygenates is favored at intermediate propylene conversions (Figure S4). Indeed, the temperature is the descriptive parameter that correlates the most with the propylene conversion, of all the candidate parameters that were offered (Figure S2). The rule on the phosphorus content shows that a relatively high phosphorus content is needed to achieve outstanding performance (Figure 2D). This could be related to the dilution of ruthenium sites on a phosphate matrix that occurs at high phosphorus loadings.^15,16^ Finally, the rule on the composition-averaged electronegativity (Figure 2F) effectively limits the range of Ru contents, as shown in the ternary diagram of Figure 3B. This reflects the fact that Ru is needed to achieve propylene conversion (Figure S5A) but that too much of this element in the composition leads to undesired combustion products (Figure S5B). The electronegativity of an element reflects its tendency to attract electronic density in a chemical bond. Thus, from a physicochemical standpoint, the relevance of  could be related to the strength and nature of certain bonds within the materials or between the materials’ surfaces and reacting species: for instance, metal–oxygen bonds.^20^ These bonds are crucial in several processes taking place during the oxidation reaction, such as such as the O_2_ dissociation and the oxygen transfers from the catalyst surface to adsorbed organic species in order to form the C–O bonds of acrolein and acrylic acid. However, we note that  is an effective (mean field) electronegativity and not a specific electronegativity of a certain element. Thus,  is also related to the composition.

We wish to stress that the rules derived by SGD depend on the *combination of constraints* on the parameters *T*, *x*_P_, and  as a descriptor for outstanding performance. Therefore, by assigning a too specific, chemical meaning to each parameter individually, one might overlook the possibly intricate interplay of the many processes governing catalysis. Moreover, the interpretation presented above is speculative in the sense that it is based on the current knowledge about the catalyst materials and reaction. It is possible, or even likely, that other, so far not well understood or unknown underlying processes (e.g., the dynamic restructuring of the catalyst during the reaction^21^) play a significant role in determining the outstanding behavior. The SG rules might capture these processes. In fact, the SG rules do not necessarily reflect causality. Thus, the physical relationship between the identified parameters and the underlying chemistry might be indirect.

Similar SG rules are obtained when the training is performed with randomly selected 90% of the data points (see cross-validation study in the Supporting Information) or when the data points presenting yield of oxygenates lower than 3% are excluded from training (see details in the Supporting Information). SG rules constraining the  parameter to an intermediate range, for instance, are always observed when only 90% of the data is used for training. Furthermore, the ranges of variation of minimum and maximum thresholds are [3.882, 3.910 eV] and [3.989, 4.031 eV], respectively: i.e., similar to the thresholds shown in Figure 2F. These results indicate that the SG rules are not strongly affected by variations of the data used for their derivation. We have also verified that the SG rules derived on the basis of 90% of the data set (training sets) are able to select the outstanding materials in the remaining 10% (test sets) (see the Supporting Information for details).

For comparison, we have also performed the SGD using only the experimental candidate descriptive parameters *T*, *x*_P_, and *x*_Ru_. We identify the SG rules 300 ≤ *T* ≤ 300 °C, *x*_P_ > 0.55, and 0.15 ≤ *x*_Ru_ ≤ 0.25. These SG rules select 13 data points that correspond to a cumulative Jensen–Shannon divergence of 0.704. The quality-function value associated with the selected data points and some of the SG rules are similar to those discussed in Figure 2. However, the rules derived solely on the basis of experimental descriptive parameters are specific to three-component materials composed of phosphorus, ruthenium, and tungsten. Thus, it is not straightforward to use these rules for the design of materials containing other elements or more than three components. Conversely, the composition-weighted parameters derived by electronic-structure calculations are well-defined for materials containing an arbitrary number of elements, including elements that are not initially present in the data set. Thus, the rules associated with the theoretical parameters can be exploited for the design of more complex materials (*vide infra*).

Overall, our results demonstrate the ability of the HTE and theory SGD approach to detect interpretable, chemically meaningful, and complex patterns associated with very few data points presenting exceptional catalytic performance.

## Exploiting the Subgroup Rules for the Design of Four- and Five-Component Catalysts

Using the rules defining the SG of outstanding oxygenate production for the three-component data, we designed more complex materials containing additional elements. We start by considering four-component materials containing ruthenium, tungsten, phosphorus, and one additional E_1_ element. For this analysis, we fix the phosphorus content to 0.60 according to the rule identified in Figure 2C. To further reduce the number of possible variables determining the catalyst composition, we also fix the ruthenium molar content to 0.05. We focus on such relatively low ruthenium loadings to ensure that the formation of combustion products is not significant. In this way, the compositions of the four-component materials are determined solely by the choice of E_1_ element and its molar content. Materials with an E_1_ molar content of 0.35, which do not contain tungsten and are thus composed by three elements, are also referred to as four-component materials in our analysis to highlight that they contain different chemical elements in comparison to ruthenium, tungsten, and phosphorus, the elements present in the materials of the data set used to derive the rules.

We concentrate on E_1_ elements that show octahedral coordination patterns among the reported phosphorus-containing material structures^22^ and that have a maximum atomic radius difference in comparison to tungsten of 0.10 Å (see details in the Supporting Information). This is to ensure that only elements that are compatible with tungsten, i.e., that could possibly replace tungsten in the material structure, are taken into account. The following E_1_ elements are considered: niobium, tantalum, chromium, molybdenum, tin, antimony, and tellurium. These elements have atomic radii of 1.45, 1.45, 1.40, 1.45, 1.45, 1.45, and 1.40 Å, respectively. The atomic radius of tungsten is 1.45 Å. We have also included lead in this analysis, since materials containing this element were also experimentally tested (see below).

**Figure 4:**
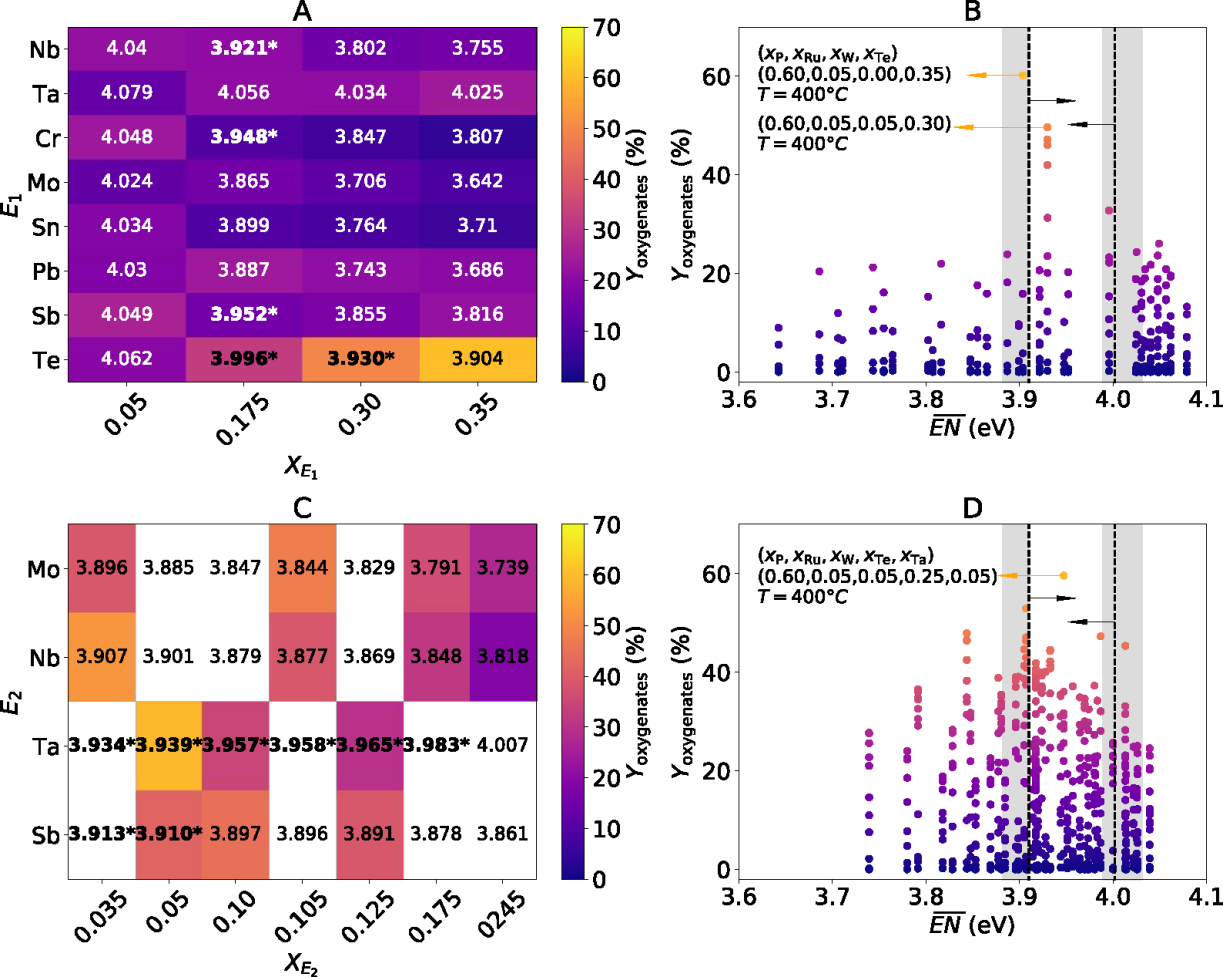
SG rules applied to the design of four- and five-component materials for propylene selective oxidation: (A, C) composition-averaged electronegativity () for different elements and molar contents in four- and five-component materials, respectively; (B, D) distribution of all measured yields of oxygenates (214 and 533 data points) for four- and five-component materials, respectively. In (A) and (C), the  values are shown in boldface and are marked with asterisks if they satisfy the SG rules on  identified on the basis of the three-component materials. The colors in (A) and (C) indicate the highest measured yield of oxygenates for each material. The SG rules identified on the basis of the three-component materials are indicated by the dashed lines and arrows in (B) and (D). The shaded areas in (B) and (D) indicate the variability of  thresholds observed when different subsets of the data, containing only 90% of the data set, are used for training (Table S2). The oxygenate yields shown in (C) correspond to materials with *x*_W_ = 0.035 for the cases E_2_ = Mo, Nb and with *x*_W_ = 0.050 for the cases E_2_ = Ta, Sb. The white cells in (C) indicate materials not measured by HTE.

We evaluated the composition-averaged electronegativity for the selected E_1_ and the molar contents 0.05, 0.175, 0.30, and 0.35 in Figure 4A. In this figure, the  values for the new four-component materials are shown in boldface and marked with asterisks if they satisfy the SG rule . This *catalyst map* suggests that the use of 5.0–17.5 mol % of niobium, chromium, molybdenum, tin, lead, and antimony, in the catalyst composition in addition to ruthenium, tungsten, and phosphorus results in catalysts which are part of the identified SG. Thus, these are likely high performing materials. For the case of tantalum and tellurium, 17.5 mol % or more of these elements is needed for the resulting materials to present  values compatible with the SG.

The four-component catalyst compositions shown in Figure 4A were tested in propylene oxidation using HTE under the same reaction conditions as those used for testing the three-component materials. The highest yield of oxygenates achieved for each composition is shown by the colors in Figure 4A. A comparison of the experimental results with the SG rules on  shows that the catalyst design rules derived by SGD correctly describe the experimental trend. In particular, the materials based on niobium, chromium, molybdenum, tin, lead, and antimony achieve the highest oxygenate yields at relative lower E_1_ molar fractions in comparison to the tantalum and tellurium-based materials, in line with the optimal ranges of  values indicated by the SG rules.

All measured yields of oxygenates, corresponding to the materials shown in the catalyst map of Figure 4A tested at several temperatures, are plotted as a function of  in Figure 4B. In this figure, the SG rules on  are shown as vertical black lines and arrows. The variability of  thresholds in the SG rule with respect to the input data set is indicated by the ranges of  values in the gray shaded areas. These ranges correspond to the variations of the thresholds observed when different subsets of data, containing only 90% of the data set, are used for training (see Table S2). The catalyst achieving the highest yield of oxygenates (60.19% at 400 °C) contains a 0.35 molar fraction of tellurium as the E_1_ element and lies within the window of  values suggested by the SG rules.

We have also used the SG rules derived from the three-component materials to address five-component materials, which were tested experimentally (Figure 4C,D). For this purpose, E_1_ was fixed to be tellurium on the basis of the best four-component catalysts and molybdenum, niobium, tantalum, and antimony were evaluated as E_2_. Thus, ruthenium, tungsten, phosphorus, tellurium, and E_2_ enter in the composition of the considered five-component materials. The agreement between the SG rule and the measured oxygenate yield is reasonable in spite of the much higher material complexity with respect to the catalysts used for training. In particular, the five-component catalyst corresponding to the highest yield of oxygenates (59.60% at 400 °C) contains tantalum as the E_2_ element and the composition-averaged electronegativity for this material is 3.947 eV. Such an  value lies within the threshold defined by the SG rule (Figure 4D).

These results demonstrate the potential of the SGD-HTE and theory approach to identify generalizable rules describing exceptional performance. Indeed, the identified four- and five-component catalysts are significantly more complex than those of the training data set (three-component materials). Moreover, the outstanding four- and five-component catalysts achieve oxygenate yields (60.19 and 59.60%, respectively) up to twice as large as those obtained with three-component materials (highest value of 26.85%). Therefore, the SG rules hinted at materials that are significantly better performing that any of the materials used in training.

We note that the four- and five-component materials achieve the highest yields of oxygenates at higher temperatures (400 °C) than the three-component systems (300 °C) (see Figure S4). One of the SG rules on the reaction temperature derived on the basis of the three-component materials data set (*T* ≤ 300 °C) is thus not transferable to the four-component set, since this aspect is not included in the training. The temperature is, however, a less crucial parameter than the composition-related parameters within our HTE approach, since the screening of different temperatures is less resource consuming than the screening of different materials, or compositions. For this reason, the four- and five-component materials were tested using the same range of temperatures used for the three-component material (200–400 °C).

**Figure 5:**
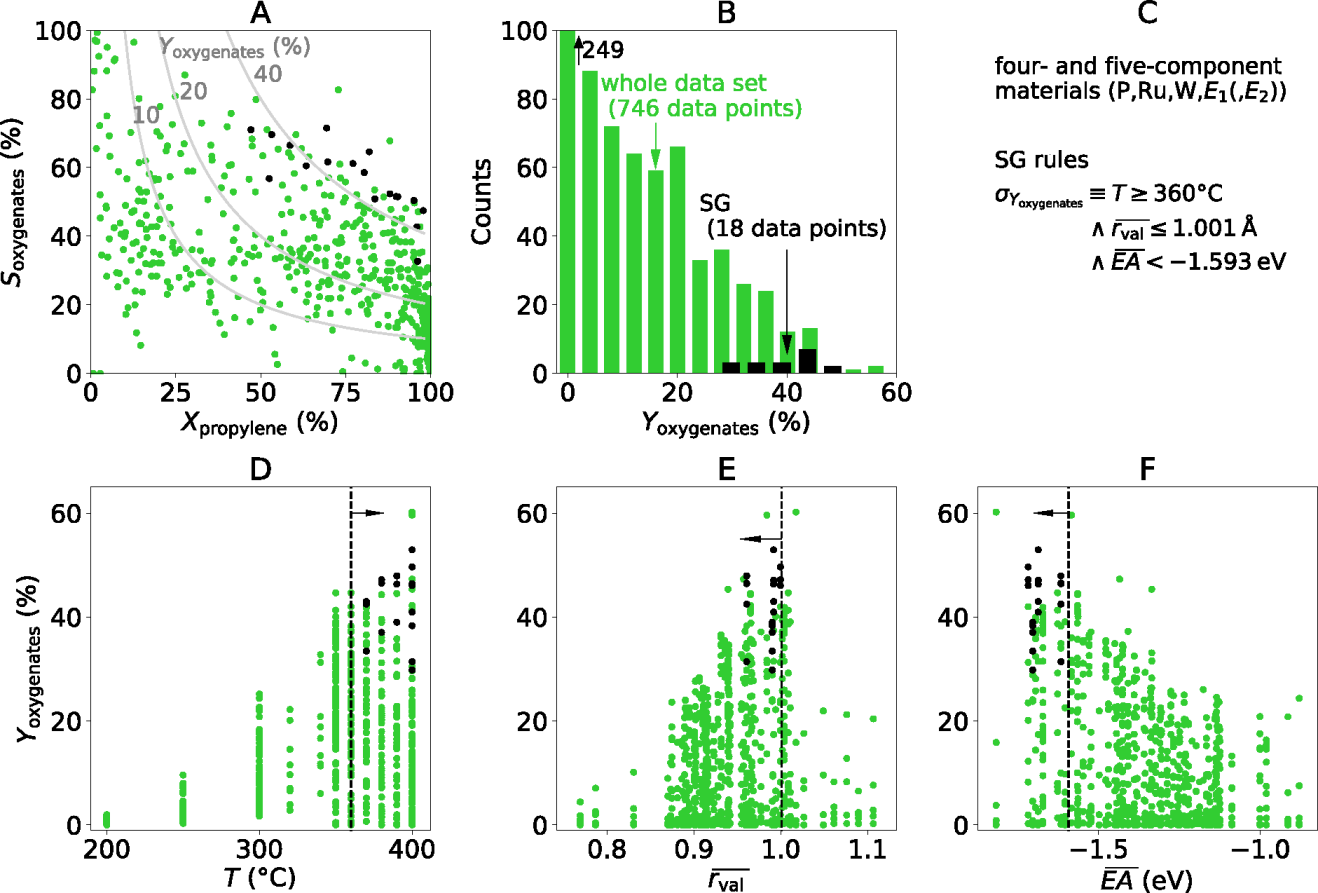
SGD analysis of propylene selective oxidation on four- and five-component materials: (A) overview of reactivity measured by HTE; (B) distribution of oxygenate yield over the data set of 746 measurements; (C) identified rules describing the SG; (D–F) SG rules (indicated by the black dashed lines and arrows) on the identified key descriptive parameter: temperature (*T*), composition-averaged valence radius (), and electron affinity (), respectively. The data points corresponding to the SG are displayed in black.

Finally, we applied the SGD approach to the four- and five-component HTE data (746 data points, Figure 5A,B) using the same candidate descriptive parameters used for the previous SGD analysis of three-component materials. The identified SG presenting the highest *D*_cJS_ value (0.355) contains 18 data points: i.e., ca. 2.4% of the data set (black points in Figure 5A,B). The selected data points correspond to one four-component material with tellurium as the E_1_ element as well as different compositions of five-component materials with E_1_ = Te and E_2_ = Mo, Nb. The rules describing this SG (Figure 5C) constrain the values of three parameters: *T* ≥ 360 °C, , and , (Figure 5D–F, respectively). The comparison of these SG rules with those for the SG obtained with the three-component materials data set (Figure 2C) highlights the higher temperatures needed for the four- and five-component materials to achieve outstanding performance. Moreover, different composition-dependent parameters ( and ) are required to describe this SG in comparison to the case of three-component materials (*x*_p_ and ), even though the electronegativity and the electron affinity are related by .

We note that two data points presenting high yield of oxygenate are not captured by the SG rules in Figure 5. This could indicate that these two data points are governed by different underlying processes in comparison to the situations belonging to the identified SG. However, these two points fall close to the threshold of the SG rules (Figure 5E,F) and the precise thresholds present some variability with respect to the data used for their derivation (see discussion above on  thresholds).

The SG rules derived in this study are expected to describe outstanding materials whose performance is governed by the same processes governing the reactivity of the exceptional materials in the input data sets used for training. The analysis of four- and five-component materials was focused, nevertheless, on low ruthenium contents and on E_1_ and E_2_ elements compatible with tungsten: i.e., with similar atomic radii. Thus, it is unclear if the SG rules presented in Figure 5C can identify exceptional materials and conditions for any arbitrary ruthenium content or for E_1_ and E_2_ elements that have significantly different radii in comparison to tungsten. This is because different mechanisms may operate on these materials that could also lead to exceptional performance. Therefore, the SGD analysis might need to be performed by including new data points covering such thus far unexplored portions of the materials space to enlarge the domain in which the SG rules can detect exceptional catalysts and reaction conditions.

## Conclusions

In this paper, we applied the SGD approach to the design of selective oxidation phosphorus-containing supported catalysts on the basis of data from HTE and DFT calculations. The yield of value-added oxygenate product measured by HTE was used as a target, and parameters obtained from DFT-calculated free-atom properties were offered as candidate descriptive parameters. The composition-weighted electronegativity, the phosphorus content, and the temperature were identified as key parameters associated with an outstanding production of acrolein and acrylic acid from propylene in three-component catalysts containing ruthenium, tungsten, and phosphorus. The SG rules on these key parameters not only rationalize a local reactivity pattern particularly associated with exceptional catalytic performance but also guide the design of more complex catalysts. In particular, a five-component material containing ruthenium, tungsten, phosphorus, tellurium, and tantalum in the composition, which presents an oxygenate yield more than twice as large as any observation in the data set used for training, is captured by the SG rules. This local modeling approach is suitable for the search of exceptional materials whose structures and functions can hardly be modeled explicitly by theory.

## Supporting Information Available

DFT calculation details, additional SGD details, and details on the choice of compatible elements for the four- and five-component materials (PDF)
High-throughput-experimentation data set (XLSX)
