# Supplementary material for: Learning Design Rules for Selective Oxidation Catalysts from High-Throughput Experimentation and Artificial Intelligence
Source: ACS Catal. 2022 Jan 31;12(4):2223–32. doi: 10.1021/acscatal.1c04793 (PMC8862133; doi:10.1021/acscatal.1c04793)
Supplement: Supplementary file 1 — cs1c04793_si_001.pdf [file cs1c04793_si_001.pdf]

Supporting Information (SI)

Learning design rules for selective oxidation catalysts from high-throughput experimentation and artificial intelligence

Lucas Foppa,[a,b*] Christopher Sutton,[a†] Luca M. Ghiringhelli,[a,b] Sandip De,[c*] Patricia Löser,[d] Stephan A. Schunk,[c,d] Ansgar Schäfer,[c] and Matthias Scheffler [a,b]

[a]The NOMAD Laboratory at the Fritz-Haber-Institut der Max-Planck-Gesellschaft, Faradayweg 4-6, D-14195 Berlin, Germany. [b]The NOMAD Laboratory at the Humboldt-Universität zu Berlin, Zum Großen Windkanal 6, D-12489 Berlin, Germany, [c]BASF SE, Carl-Bosch-Straße 38, D-67065 Ludwigshafen, Germany. [d]hte GmbH, Kurpfalzring 104, D-69123, Heidelberg, Germany. *foppa@fhi-berlin.mpg.de, sandip.de@basf.com

Density-functional theory calculations

The elemental free-atom properties were calculated with the FHI-aims full-electron full-potential code[1] using the PBEsol exchange-correlation functional[2] and "really tight" basis set and settings. The self-consistent cycle is considered converged when the change in charge density is less than 10^{-5}, the change in the sum of the eigenvalues is less than 10^{-3}, and the change in the total energy is less than 10^{-5}. All other settings were taken as default in FHI-aims. r_s, and r_{val} are defined as the radius of the valence *s* and the highest occupied state, respectively. These radii also correspond to the maximum and minimum radii of the filled valence shell of an atom. The highest occupied and lowest unoccupied states are defined based on the partial occupancy of the electronic states. EA and IP are calculated by the energy difference between the neutral and charged systems. The values of the elemental properties for the elements considered in our study are shown in Table S1.

Subgroup-discovery approach

The starting point for the SGD approach is a data set P with each data point corresponding to a different material and/or applied external condition. For each of the data points, the value of a *target* of interest, e.g., a materials property or function, as well as the values for several *candidate descriptive parameters*, $\varphi_1, \ldots, \varphi_N$, , are known. The candidate descriptive parameters are physicochemical parameters describing the materials and external conditions that are potentially related to the processes governing the target. From this data set, the SGD approach identifies subsets of data, i.e., *subgroups*, which present an outstanding distribution of the target values. The distribution of the target values in the SG might be outstanding because it is, for instance, narrower (i.e., it has lower standard deviation) or shifted, towards lower or larger values, with respect to the whole data set. The function measuring how outstanding a SG is with respect to the whole data set is called *quality function*.

The SG search is performed in two steps. Firstly, a number of selectors, i.e., combination of statements about the data, are generated. These conjunctions are Boolean functions having the form:

$$\sigma(\varphi) \equiv c_1(\varphi) \wedge c_2(\varphi) \wedge \ldots \wedge c_p(\varphi), \qquad \text{(S1)}$$

where "$\wedge$" denotes the "AND" operator and each statement π_i, referred to as *proposition*, is an inequality constrain on one of the descriptive parameter, for instance

$$\pi_i(\varphi) \equiv \varphi_i \geq v_i \text{ or } \pi_j(\varphi) \equiv \varphi_j < v_j, \qquad \text{(S2)}$$

for some constant v_i to be determined in the analysis. The selectors describe simple convex regions ($\{\varphi \in \phi: \sigma(\varphi) = \text{true}\}$) in the descriptive parameter space ϕ which define SGs of the data set. To keep the number of v_i values computationally tractable, a finite set of cut-offs is determined using *k*-means clustering. Secondly, a search algorithm is employed to identify SGs defined by the generated selectors that maximize the quality function. The outcome of this search is a list of SGs ranked according to the quality-function values. The most relevant SGs are those presenting the highest quality-function values. The selectors defining each of these SGs depend on key descriptive parameters associated to the outstanding behavior in each subset of materials. The propositions entering the selectors can be seen as *rules* determining the outstanding SG performance.

SGD is a supervised descriptive rule-induction technique, since it uses the labels assigned to the data points, i.e., the target values, to identify patterns in the descriptive parameter space. Furthermore, SGD is based on the maximization of a function that focuses on specific subselections of the dataset. For this reason, it is suitable to detect exceptional *local* types of behavior. This contrasts with artificial-intelligence methods such as decision-tree regression, which are based on the minimization of the error across the whole data set and thus provide a description of the *global* behavior. Although global-modelling approaches are suitable to understand the general trend, they might fail in detecting statistically exceptional, interesting regions of the materials space for which only few observations are available. This is because these regions of the materials space do not significantly impact the optimized loss function, and thus the final model.

In our SGD analysis, we use D_{cJS} the cumulative-distribution function formulation of D_{JS}, the Jensen-Shannon divergence, to quantify, along with the coverage term (see Eq. 3), how outstanding a SG is. D_{JS} is a measure of dissimilarity between two distributions (e.g., P and P') defined by

$$D_{\mathrm{JS}}(P||P') = \frac{1}{2}D_{\mathrm{KL}}(P||M) + \frac{1}{2}D_{\mathrm{KL}}(P'||M), \qquad \text{(S3)}$$

where $M = \frac{P+P'}{2}$ and D_{KL} is the Kulback-Leibler divergence. Thus, the D_{JS} is a symmetrized version of D_{KL} and the same divergence value is obtained irrespective of the choice of P and P'. D_{KL} is defined, in the case of discrete distributions, by

$$D_{\mathrm{KL}}(P||P') = \sum_{x\epsilon\chi} P(x)\, log\, \frac{P(x)}{P'(x)}, \qquad \text{(S4)}$$

where χ indicates the probability space. D_{KL} is also called relative entropy, due to the similarity of its expression with the Shannon entropy for a random variable X:

$$H(X) = -\sum_{x\epsilon\chi} P(x) log P(x). \qquad \text{(S5)}$$

In order to get an intuition on how the D_{JS} influences the SGD approach, we evaluated the D_{JS} between a fixed normal distribution P (shown in orange in Fig. S1) and several normal distributions P' (shown in black in Fig. S1) whose mean value or narrowness were modified with respect to the P. In the context of SGD P can be seen as the distribution of the target over the whole data set, whereas P' is analogous to the several possible SGs of the data set. The narrowness corresponds to the standard deviation of the distributions. Figure S1 shows that when P and P' are the exact same distribution, D_{JS} is equal to zero. The more P' mean value is shifted with respect to P, the highest the D_{JS} gets (Fig S1, horizontal panels). Similarly, the narrower P' is with respect to P, the highest the D_{JS} gets (Fig S1, vertical panels). Because the quality function is *maximized* during the SG search, the more shifted and narrow the SG is, the more outstanding it will be considered. When most of the materials in the data set have poor performance, the use of D_{JS} in the quality function favors the identification of SG containing the few materials with high performance, i.e., "the needles on the haystack". Finally, D_{cJS} is used in our SGD approach because it can be efficiently calculated from the data without the need for estimations.

We performed the SGD analysis using the CREEDO code version 0.5.1 as implemented in realkd.[3] In order to generate the propositions about the data, we used a *k*-means clustering with 20 clusters. The "randomized exceptional subgroup discovery" algorithm based on a Monte-Carlo search was used for the SG search. In spite of the stochastic nature of the search algorithm, we highlight that the quality-function values for each identical SGs will always assume the same value. The outcome of the SGD approach is a list of SGs ranked by their quality-function values. Based on the same data set and SGD settings, different SGs can be identified presenting near-optimal quality-function values. In this work, we took into account SGs presenting quality-function values within 40% of the maximum observed (optimum) value. The diversity of SGs that can be identified with similar quality-function values is illustrated in Fig. S3. These SG are often defined by similar selectors, i.e., with similar descriptive parameters and thresholds, but they present rather different distributions of target values. We focused the discussion on the identified SG with the highest D_{cJS} values - and rather low coverage – among all identified SGs with near-optimal quality-function values because thes SGs contain exclusively materials and conditions with exceptional performance. However, we note that other SGs with higher coverage and lower D_{cJS} values also present similar quality-function value, see for instance the SGs shown in Fig. S3. It should be also observed that some of the candidate descriptive parameters might be significantly correlated with each other (Fig. S2). Thus, the same subselection of data points (and therefore the same quality-function value) can be obtained by using different descriptive parameters and thresholds.

We have also applied the SGD approach to subselections of the three-component materials data set to assess the variability of the SG rules with respect to the data used for training and to verify the performance of the SG rules on unseen three-component materials data. For this purpose, we split the data set (1220 data points) according to a 10-fold cross-validation split, i.e., the data set is split in 10 portions and each of these portions is excluded from training at a time. Thus, the SGD is applied to *training data sets* containing 1098 data points and the excluded portions (122 data points) are used as *test sets*. The SG rules obtained using this scheme and using the yield of oxygenates as target (Table S2), constrain, in all cases, the descriptive parameters T and $\overline{EN}$ to intermediate ranges. Additionally, either x_{P}, $\overline{EA}$, $\overline{\epsilon_{\mathrm{L}}}$, $\overline{r_d}$, or $\overline{r_s}$ appear in one additional rule, depending on the training data used. We note that $\overline{EA}$, $\overline{\epsilon_{\mathrm{L}}}$, $\overline{r_d}$, or $\overline{r_s}$ are all significantly correlated with x_{P}. The absolute (pairwise) Pearson correlation score between each of these parameters with x_{P} is higher than 0.92 - see Fig. S2A. Thus, the key parameters shown in Table S2 can be considered equivalent to those discussed in Fig. 2 (T, $\overline{EN}$ and x_{P}). Furthermore, in most of the cases the thresholds entering the SG rules obtained by training with 90% of the data set are similar to those obtained with the whole data set (Fig. 2C). In particular, the lower and higher bounds for $\overline{EN}$ are in the ranges [3.882, 3.910 eV] and [3.989, 4.031 eV], respectively, while the respective values obtained with the whole data set are 3.910 and 4.002 eV, respectively. These results indicate that the SG rules are not significantly affected by the input data used for their derivation. Finally, the average yield of oxygenates calculated on the test-set points selected by the SG rules are much higher than the average value of $Y_{\mathrm{oxygenates}}$ calculated over the entire test sets (Table S2), indicating that the SG rules effectively identify the exceptional materials on unseen three-component materials data.

Finally, we also applied the SGD to a reduced data set of three-component catalysts, from which the data points providing less than 3% yield of oxygenate were excluded. This reduced data set contains 519 data points. Among the identified SGs with near-optimal quality-function values, the SG corresponding to the largest Janson-Shannon divergence (0.638) contains 12 data points and it is described by the selector $300 \leq T \leq 305℃ \wedge \overline{EN} < 4.002\ \mathrm{eV} \wedge x_{\mathrm{P}} > 0.55$. Such conjunction is similar to that discussed in Fig. 2, with the exception that it does not impose a lower bound for $\overline{EN}$. Therefore, the SG rules are not significantly affected by the exclusion of the low-performant materials from the data set used for training.

Choice of compatible E_1 and E_2 elements for the four- and five-component materials

In order to select compatible elements in four- and five-component materials, we looked at the HT4CAT database[4] and searched for structures containing phosphorus and oxygen elements in their composition. Among the elements present on these structures, we selected those showing octahedral coordination and a maximum atomic radius difference compared to tungsten of 0.10 Å. This resulted in the elements shown in Table S4.

Table S1. Elemental properties used to derive the composition-dependent candidate descriptive parameter. These are free-atom properties evaluated using DFT-PBEsol and the FHI-aims code.

element	r_s (Å)	r_p (Å)	r_d (Å)	r_{val} (Å)	ϵ_H (eV)	ϵ_L (eV)	EA (eV)	IP (eV)	EN (eV)
Ru	1.4758	1.5475	0.6474	0.6474	-3.5656	-3.5656	-0.9258	7.6733	3.3738
W	1.4053	1.6055	0.7908	0.7908	-4.4642	-4.4642	-0.4082	8.8159	4.2038
P	0.8343	0.9719	1.4171	0.9719	-5.4577	4.2424	-1.5547	9.7140	4.0797
Nb	1.5761	1.7315	0.7736	0.7736	-4.4928	-4.4928	-0.6607	7.1652	3.2522
Ta	1.4650	1.6635	0.8373	0.8373	-3.5407	-3.5407	0.2861	7.7633	4.0247
Cr	1.4247	1.4064	0.4394	0.4393	-4.3916	-4.3916	-0.5037	7.3085	3.4024
Mo	1.5378	1.6614	0.7275	0.7275	-4.2617	-4.2617	-1.1447	7.0073	2.9313
Sn	1.0659	1.3540	0.4696	1.3540	-3.6033	-3.6033	-0.6245	6.8758	3.1257
Pb	1.0397	1.4029	0.5678	1.4029	-3.3881	-3.3881	-0.4610	6.5720	3.0555
Sb	1.0033	1.2387	0.4484	1.2386	-4.7867	5.0023	-1.4817	8.3375	3.4279
Te	0.9494	1.1484	0.4294	1.1484	-5.9351	4.9135	-2.3924	9.7470	3.6773

Table S2. SGs of three-component materials presenting outstanding yield of oxygenates obtained using 90% of the data set as training set (1098 data points). The performance of the SG rules on the test sets (122 data points) is also shown in the table.

#data points in SG, training	D_{cJS}	SG rules obtained with the training set	Test-set $\overline{Y_{\mathrm{oxygenates}}}$ (%)	Test-set-SG $\overline{Y_{\mathrm{oxygenates}}}^{a}$ (%)	# data points in SG, test
16	0.562	$300 \leq T \leq 300℃ \wedge 3.910 \leq \overline{EN} \leq 4.031\ \mathrm{eV} \wedge \overline{EA} < -1.127\mathrm{eV}$	6.45	24.00	3
13	0.694	$275 \leq T \leq 300℃ \wedge 3.910 \leq \overline{EN} \leq 3.989\ \mathrm{eV} \wedge \overline{\epsilon_L} > 0.661\ \mathrm{eV}$	3.58	-	0^b
12	0.719	$290 \leq T \leq 300℃ \wedge 3.910 \leq \overline{EN} \leq 4.002\ \mathrm{eV} \wedge x_P > 0.55$	4.81	-	0^b
13	0.696	$267.5 \leq T \leq 300℃ \wedge 3.882 \leq \overline{EN} \leq 3.989\ \mathrm{eV} \wedge x_P > 0.55$	4.63	-	0^b
12	0.665	$280 \leq T \leq 300℃ \wedge 3.882 \leq \overline{EN} \leq 4.002\ \mathrm{eV} \wedge \overline{\epsilon_L} > 0.616\ \mathrm{eV}$	4.91	26.10	3
15	0.568	$255 \leq T < 300℃ \wedge 3.882 \leq \overline{EN} < 3.989\ \mathrm{eV} \wedge x_P > 0.55$	4.21	11.60	1
15	0.490	$255 \leq T \leq 300℃ \wedge 3.910 \leq \overline{EN} < 4.002\ \mathrm{eV} \wedge \overline{r_d} > 1.11$ Å	5.50	24.20	3
14	0.652	$300 \leq T \leq 300℃ \wedge 3.903 \leq \overline{EN} < 3.989\ \mathrm{eV} \wedge \overline{r_s} < 1.133$ Å	4.94	-	0^b
15	0.542	$280 \leq T \leq 300℃ \wedge 3.910 \leq \overline{EN} < 3.989\ \mathrm{eV} \wedge x_P > 0.525$	4.65	20.97	4
13	0.733	$280 \leq T \leq 300℃ \wedge 3.882 \leq \overline{EN} < 4.002\ \mathrm{eV} \wedge x_P > 0.55$	4.64	25.80	2

[a]Average of oxygenate yield calculated over the test-set data points selected by the SG rules. [b]No test data point is selected by the SG rules. The average oxygenate yield over the whole data set of three-component materials (1220 data points) is equal to 4.83% and the maximum value is 26.85 %.

Table S3. Compatible E_1 and E_2 elements used in four- and five-component materials

element	atomic radius (Å)	common oxidation states
Os	1.30	4
Pt	1.35	2,4
Re	1.35	4
Ir	1.35	3,4
Ru	1.30	3,4
Te	1.40	-2,2,4,6
Ta	1.45	5
Sn	1.45	-4,2,4
Sb	1.45	-3,3,5
Cr	1.40	3,6
W	1.35	4,6
Nb	1.45	5
V	1.35	5
Mo	1.45	4,6
Mn	1.40	2,4,7
Ti	1.40	4

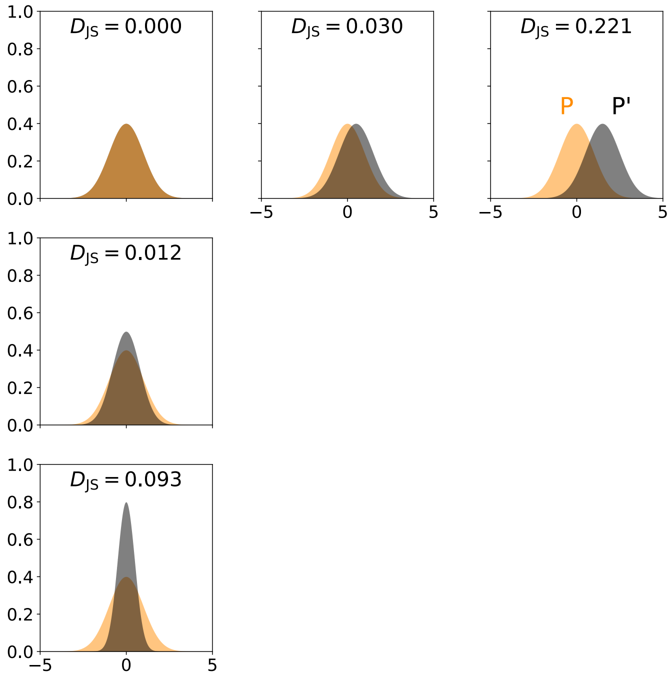

Figure S1. Jensen-Shannon divergence values calculated for different normal distributions. (horizontal panels) D_{JS} between normal distributions with the same narrowness but different mean values. (vertical panels) D_{JS} between normal distributions with the same mean values but different narrowness. The narrowness corresponds to the standard deviation of the distributions.

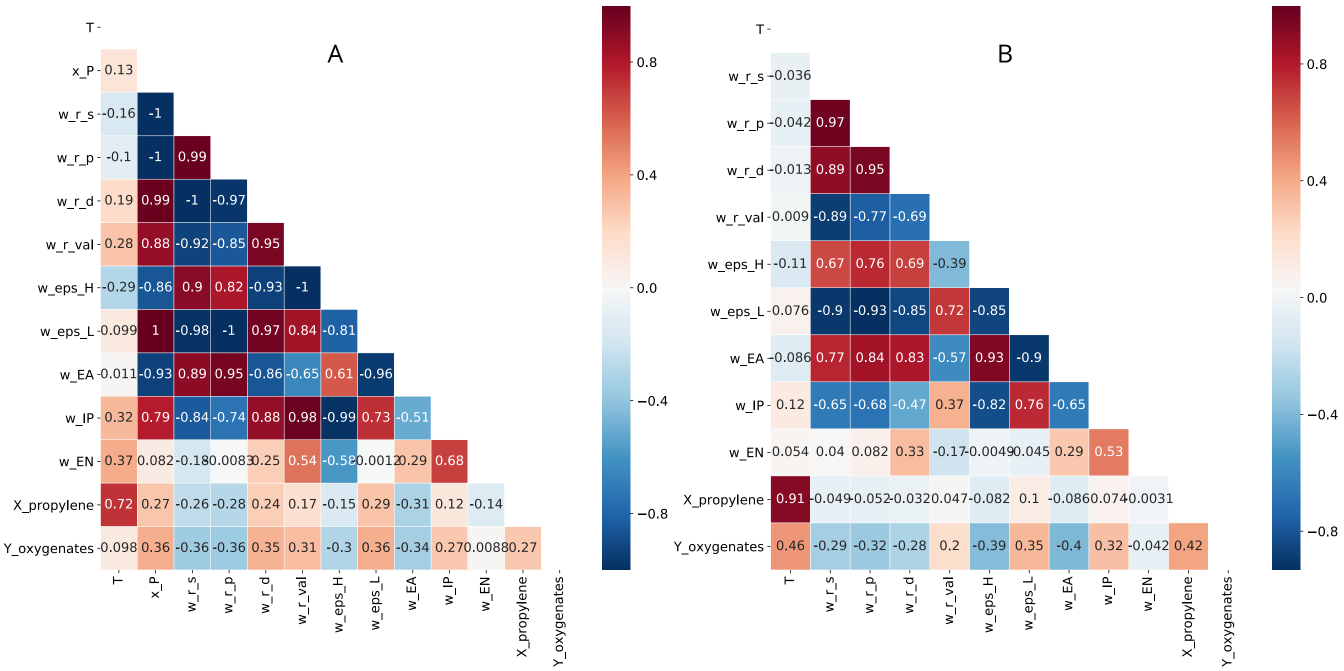

Figure S2. Pearson correlation among the candidate descriptive parameters. A: three-component materials data set (1220 data points) and B: four- and five-component materials data set (746 data points). The color scale indicates the values of the correlation score, which are explicitly shown in the graph. In the labels associated to candidate descriptive parameters, "w" indicates composition-weighted averages.

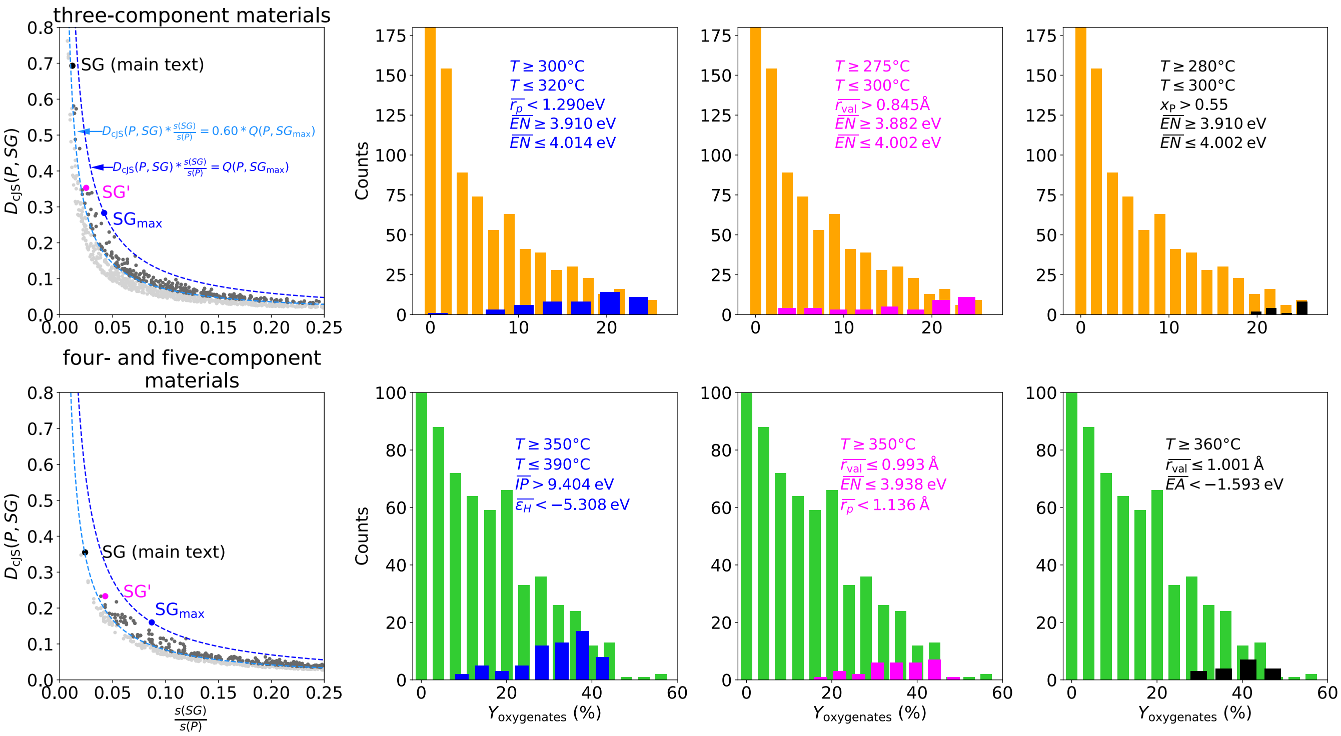

Figure S3. Diversity of SGs identified for the target yield of oxygenates in propylene selective oxidation using the three-component materials data set (1220 data points, top in orange) as well as the four- and five-component materials data set (764 data points, bottom in green). In addition to the SGs discussed in the main text (indicated in black), other SGs with lower cumulative Jensen-Shannon divergence values and higher coverages but similar quality-function values are obtained via SGD (left). The distribution of target values for some other identified SGs and the selectors describing these SGs are shown in the panels on the right.

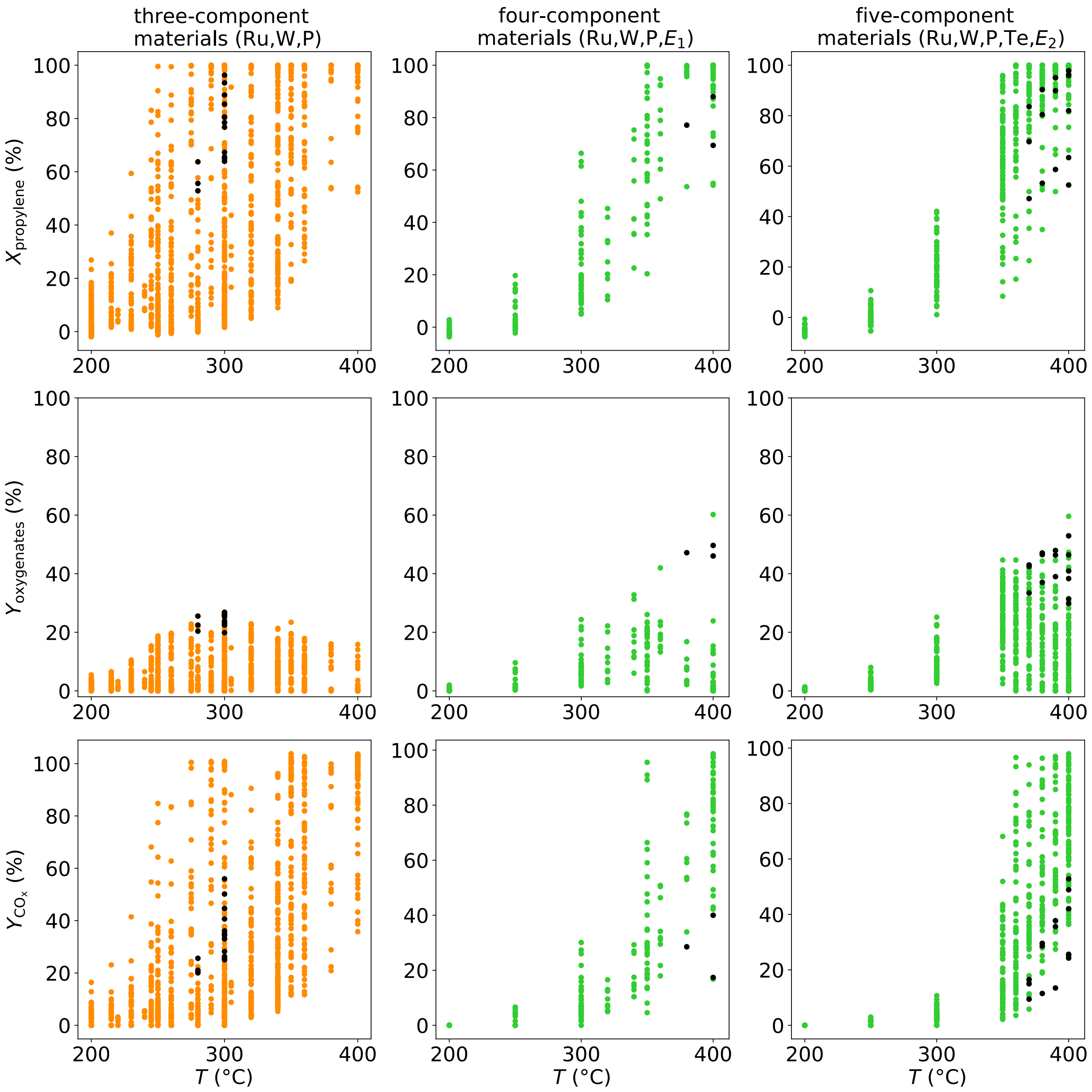

Figure S4. Temperature-dependence on propylene conversion and product yield achieved by the catalysts analyzed by HTE in this work. The data points corresponding to the identified SGs (using three-component or four- and five-component material datasets) are shown in black. The propylene conversion is defined by $\frac{\dot{F}_{propylene,in}-\dot{F}_{propylene,out}}{\dot{F}_{propylene,in}}$. The CO_x product yield is defined by $\frac{\dot{F}_{CO,out}+\dot{F}_{CO_2,out}}{3\dot{F}_{propylene,in}}$.

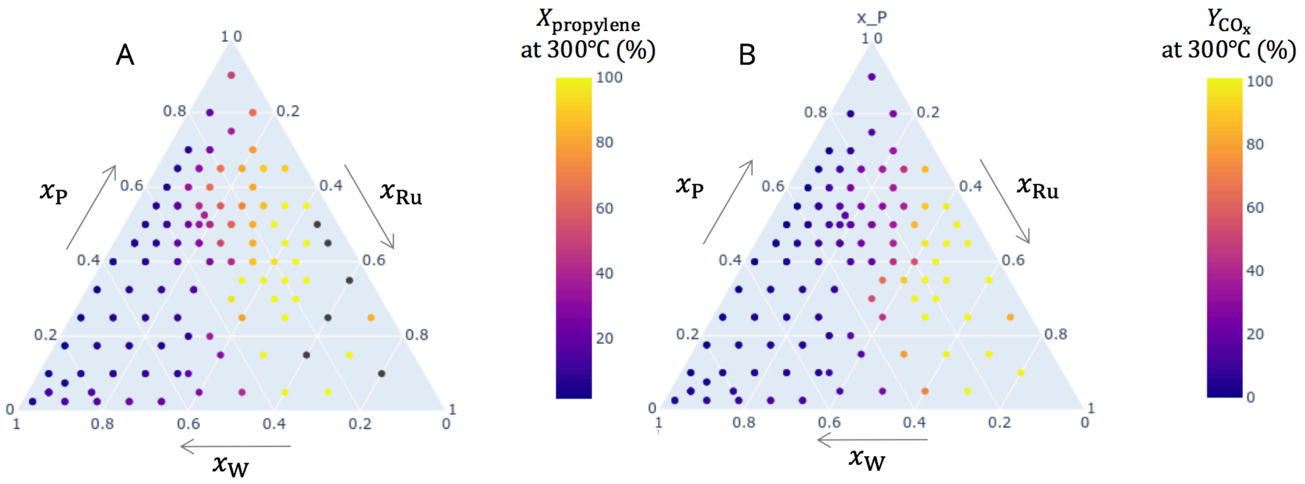

Figure S5. Ternary diagrams for three-component materials with ruthenium, tungsten, and phosphorus tested in propylene selective oxidation using HTE showing the performance measured at 300° C. A: Propylene conversion (defined by $\frac{\dot{F}_{\text{propylene,in}}-\dot{F}_{\text{propylene,out}}}{\dot{F}_{\text{propylene,in}}}$). B: $\mathrm{CO_x}$ product yield (defined by $\frac{\dot{F}_{\text{CO,out}}+\dot{F}_{\text{CO}_2\text{,out}}}{3\dot{F}_{\text{propylene,in}}}$).

References

1. V. Blum, R. Gehrke, F. Hanke, P. Havu, V. Havu, X. Ren, K. Reuter and M. Scheffler, *Ab initio* molecular simulations with numeric atom-centered orbitals. Comput. Phys. Commun. 180, 2175 (2009).
2. G.I. Csonka, J.P. Perdew, A. Ruzsinszky, P.H.T. Philipsen, S. Lebègue, J. Paier, O.A. Vydrov and J.G. Ángyán, Assessing the performance of recent density functionals for bulk solids. Phys. Rev. B 79, 155107 (2009).
3. CREEDO is a web application that provides an intuitive graphical user interface for real knowledge discovery algorithms and allows to rapidly design, deploy, and conduct user studies. See http://realkd.org/creedo-webapp/for additional information. See also the NOMAD analytics-toolkit for a tutorial.
4. D. Waroquiers, X. Gonze, G.-M. Rignanese, C. Welker-Nieuwoudt, F. Rosowski, M. Göbel, S. Schenk, P. Degelmann, R. André, R. Glaum and G. Hautier, Statistical Analysis of Coordination Environments in Oxides. Chem. Mater. 29, 8346 (2017).
